# Cardiac dysfunction and high-sensitive C-reactive protein are associated with troponin T elevation in ischemic stroke: insights from the SICFAIL study

**DOI:** 10.1186/s12883-022-03017-1

**Published:** 2022-12-31

**Authors:** Felipe A. Montellano, Elisabeth J. Kluter, Viktoria Rücker, Kathrin Ungethüm, Daniel Mackenrodt, Silke Wiedmann, Tassilo Dege, Anika Quilitzsch, Caroline Morbach, Stefan Frantz, Stefan Störk, Karl Georg Haeusler, Christoph Kleinschnitz, Peter U. Heuschmann

**Affiliations:** 1 Institute of Clinical Epidemiology and Biometry, University of Würzburg, Würzburg, Germany; 2 Interdisciplinary Center for Clinical Research, University Hospital Würzburg, Würzburg, Germany; 3 Department of Neurology, University Hospital Würzburg, Würzburg, Germany; 4 Comprehensive Heart Failure Center, University and University Hospital Würzburg, Würzburg, Germany; 5 Department of Internal Medicine I, University Hospital Würzburg, Würzburg, Germany; 6 Department of Neurology and Center for Translational and Behavioral Neurosciences (C-TNBS), University Hospital Essen, Essen, Germany; 7 Clinical Trial Center, University Hospital Würzburg, Würzburg, Germany

**Keywords:** Ischemic stroke, Troponin, Heart failure, Biomarkers, Echocardiography

## Abstract

**Background:**

Troponin elevation is common in ischemic stroke (IS) patients. The pathomechanisms involved are incompletely understood and comprise coronary and non-coronary causes, e.g. autonomic dysfunction. We investigated determinants of troponin elevation in acute IS patients including markers of autonomic dysfunction, assessed by heart rate variability (HRV) time domain variables.

**Methods:**

Data were collected within the Stroke Induced Cardiac FAILure (SICFAIL) cohort study. IS patients admitted to the Department of Neurology, Würzburg University Hospital, underwent baseline investigation including cardiac history, physical examination, echocardiography, and blood sampling. Four HRV time domain variables were calculated in patients undergoing electrocardiographic Holter monitoring. Multivariable logistic regression with corresponding odds ratios (OR) and 95% confidence intervals (CI) was used to investigate the determinants of high-sensitive troponin T (hs-TnT) levels ≥14 ng/L.

**Results:**

We report results from 543 IS patients recruited between 01/2014–02/2017. Of those, 203 (37%) had hs-TnT ≥14 ng/L, which was independently associated with older age (OR per year 1.05; 95% CI 1.02–1.08), male sex (OR 2.65; 95% CI 1.54–4.58), decreasing estimated glomerular filtration rate (OR per 10 mL/min/1.73 m^2^ 0.71; 95% CI 0.61–0.84), systolic dysfunction (OR 2.79; 95% CI 1.22–6.37), diastolic dysfunction (OR 2.29; 95% CI 1.29–4.02), atrial fibrillation (OR 2.30; 95% CI 1.25–4.23), and increasing levels of C-reactive protein (OR 1.48 per log unit; 95% CI 1.22–1.79). We did not identify an independent association of troponin elevation with the investigated HRV variables.

**Conclusion:**

Cardiac dysfunction and elevated C-reactive protein, but not a reduced HRV as surrogate of autonomic dysfunction, were associated with increased hs-TnT levels in IS patients independent of established cardiovascular risk factors.

Registration-URL: https://www.drks.de/drks_web/; Unique identifier: DRKS00011615.

**Supplementary Information:**

The online version contains supplementary material available at 10.1186/s12883-022-03017-1.

## Background

Cardiac troponin levels are sensitive and specific markers of myocardial injury [1] and are routinely used to diagnose myocardial infarction [1]. Troponin elevation can also be observed in 20–60% of patients with acute ischemic stroke (IS) and has been associated with IS of cardioembolic origin [2], poor functional outcome [3], and increased short- and long-term mortality [4, 5]. The current American Heart Association guideline for the early management of patients with acute IS recommends routine troponin measurement [6], although the independent association of troponin with poor outcome is inconsistent [7].

Troponin elevation may be caused by a thrombotic acute coronary syndrome preceding or concomitant to acute IS. Postulated mechanisms of troponin elevation due to non-acute coronary causes comprise heart failure [8, 9], impaired kidney function [8], and autonomic dysfunction due to sympathovagal imbalance with a dominating sympathetic activation [3, 10].

Comparability of prevalence and determinants of troponin elevation in IS patients between existing studies [3, 8–12] is limited due to heterogeneous inclusion criteria and the variety of troponin assays employed, with differing sensitivities and upper reference limits (URL). Information on the correlates of troponin elevation is often derived from studies with limited sample size, precluding adequately powered multivariable analyses [3, 11]. Further, the available evidence frequently relies on retrospective analyses of routinely collected data, where uptake of cardiac investigations is often incomplete [8–10, 12].

Markers of autonomic function (e.g., plasma catecholamines) are not routinely measured in IS patients and may be influenced by environmental stress [13]. Hence, this possible pathomechanism has not been well studied. The assessment of the heart rate variability (HRV) may provide a non-invasive and reproducible alternative to investigate the autonomic function in IS patients [14]. Time domain variables provide an estimate of the amount of HRV at various time scales, reflecting fluctuation in autonomic input to the heart. Reduced HRV may indicate autonomic dysfunction resulting from both autonomic withdrawal or saturating sympathetic input [15]. Inflammation has also been postulated as a potentially relevant pathomechanism in the heart-brain interaction [16], although the clinical evidence is scarce.

Therefore, we examined determinants of troponin elevation >99th percentile using a high-sensitivity assay within prospectively collected data undergoing detailed cardiac phenotyping at baseline including HRV time domain variables.

## Methods

Patients were recruited within the prospective Stroke Induced Cardiac FAILure in mice and men (SICFAIL) study, a hospital-based, investigator-initiated cohort intended to investigate the prevalence and natural course of cardiac dysfunction after IS (clinical trial registration: DRKS00011615). The methodology of the SICFAIL study has been previously described [17]. In brief, patients ≥18 years with IS according to the WHO definition [18] were recruited at Stroke Unit of the Department of Neurology of the Würzburg University Hospital between January 2014 and February 2017. Exclusion criteria were a final diagnosis other than IS and participation in an interventional study. All patients underwent routine diagnostic workup (for details see Additional file 1 Appendix I).

### Baseline investigation

Patients or next of kin knowledgeable about the patient’s history were interviewed about socio-demographic factors, previous medical history (for definitions see Additional file 1 Appendix I), symptoms suggestive of heart failure, and medications on admission. Stroke severity (assessed by the National Institutes of Health Stroke Scale, NIHSS), and clinical signs suggestive of heart failure were documented on admission.

### Echocardiography

Cardiac structure and function were assessed using transthoracic echocardiography by certified echocardiography technicians [19] of the Comprehensive Heart Failure Center Würzburg on a high-end ultrasound device (Vivid E9, GE Healthcare, GE M5S-D matrix single-crystal phased array 1.5–4.5 MHz transducer). A minimum of three cardiac cycles was recorded for analysis and stored digitally. Left ventricular systolic dysfunction was defined as left ventricular ejection fraction < 52% in men and < 54% in women [20]. Left ventricular diastolic function was defined as fulfillment of at least three of the following criteria: [1] left atrial volume index > 34 mL/m^2^ or left atrial area > 30 cm^2^ (if left atrial volume index not available), [2] average E/e’ > 14, [3] lateral e’ < 10 cm/s or septal e’ < 7 cm/s, [4] tricuspid regurgitation maximal flow velocity > 2.8 m/s [21]. Because diastolic and systolic dysfunction have a marked overlap [22], diastolic dysfunction was reported only in patients without systolic dysfunction, as previously described [17]. Clinically overt heart failure was defined as patient fulfilling clinical, echocardiographic, and biomarker criteria [23], as previously described [17].

### Biomarker measurement

High-sensitive troponin T (hs-TnT), high-sensitive C-reactive protein (hs-CRP), and creatinine were measured from fasting blood samples drawn at median 3 days (quartiles 2–4) after symptoms onset and stored at − 80 °C in the Interdisciplinary Bank of Biomaterials and Data Würzburg (ibdw) [24] (for further details see Additional file 1 Appendix I). Hs-TnT elevation was defined as a value >99th percentile URL (≥14 ng/L) in the assay’s validation study [25]. Estimated glomerular filtration rate (eGFR) was calculated using the Chronic Kidney Disease Epidemiology Collaboration formula [26].

### ECG Holter monitoring and HRV analysis

Patients underwent ECG Holter monitoring as part of the routine diagnostic workup using a 3-channel (12-lead) Holter ECG (Philips Zymed Digitrak XT, Eindhoven, The Netherlands). Initial inspection of QRS complexes for ectopy and artifact labelling was performed using commercially available software (Philips Zymed Holter 2010 Plus/1810 Series). Annotated beat-to-beat files underwent a second stage of manual editing of R-R intervals using dedicated software (Heart Rate Variability Analysis aHRV. Release 13.4.0, Nevrokard Kiauta, d.o.o., Izola, Slovenia). The following time domain variables were calculated in accordance with published recommendations: [15] (1) standard deviation (SD) of normal-to-normal beats (SDNN, estimate of overall HRV), (2) SD of the averages of normal-to-normal intervals for all 5-min segments for 24 h (SDANN, estimate of long-term components of HRV), (3) mean of 5-min SDs of all normal-to-normal intervals for 24 h (SDNN index, estimate of HRV due to cycles shorter than 5 minutes), (4) root mean square of successive RR interval differences (RMSSD, estimate of short-term components of HRV).

**Fig. 1:**
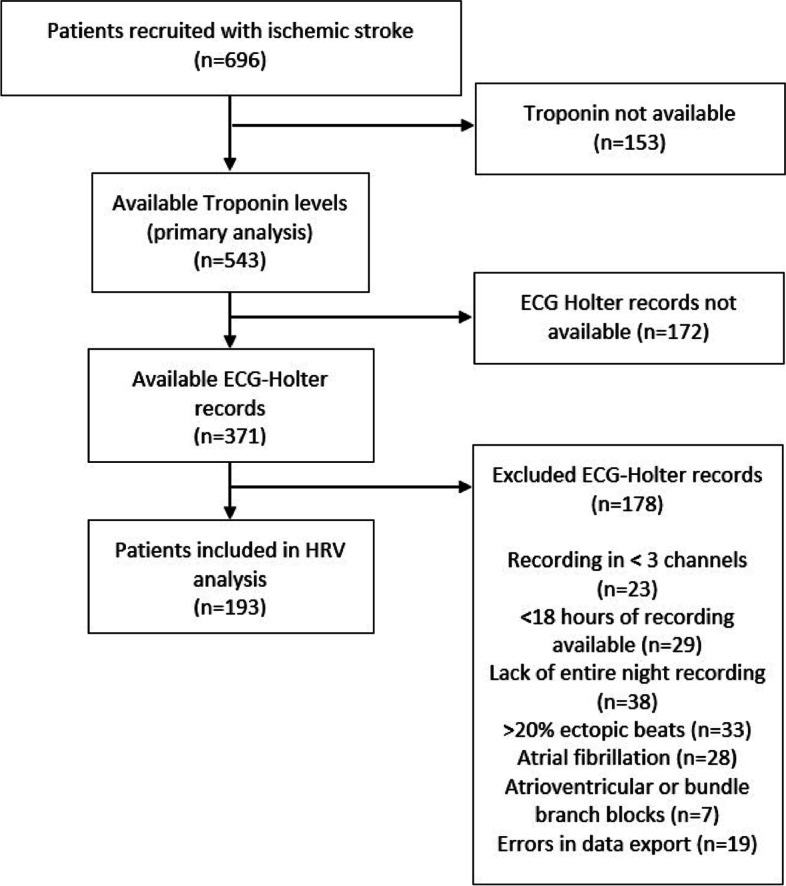
Flow diagram of study participants

Only records fulfilling the following prespecified criteria were included in this analysis: normal sinus rhythm with ≥18 hours of 5-minute segments with ≥80% normal R-R intervals and < 20% ectopic beats, recorded in all three channels and including the entire night (midnight to 6 AM). Patients with pacemaker, long segments of atrial fibrillation, and atrioventricular or bundle branch block were excluded (see Fig. 1).

### Statistical analysis

We tested differences between groups using the χ^2^ test, Student’s t-test, and Mann–Whitney U test, according to the distribution of the variables. We report frequencies for patients with complete information regarding the specified criteria. We used multivariable logistic regression analysis to determine the association of predefined variables with a troponin level ≥ 14 ng/L and report odds ratios (OR) with 95% confidence intervals (CI). The variables of interest were selected a priori based on background clinical knowledge: age, sex, systolic dysfunction, diastolic dysfunction in absence of systolic dysfunction, atrial fibrillation, eGFR, history of coronary artery disease (CAD), severity (assessed by the NIHSS), insular stroke, and hs-CRP. Collinearity was investigated using the variance inflation factor. Concentrations of hs-CRP were logarithmically transformed due to skewed distribution. In a sensitivity analysis, we investigated: (1) the consistency of the association of the previously selected variables with troponin elevation after adjusting for atherogenic cardiovascular risk factors (diabetes mellitus, hypercholesterolaemia and low-density lipoprotein levels, smoking, and hypertension); and (2) the association of single HRV time domain variables with hs-TnT ≥14 ng/L in patients with available HRV data. Due to the limited amount of events, we used a parsimonious model for the second sensitivity analysis and adjusted for age (model 1), or age and eGFR (model 2), based on previous data identifying age and eGFR as the single most relevant variables associated with hs-TNT ≥14 ng/L in non-coronary patients [27]. HRV variables (SDNN, SDANN, SDNN index, RMSSD) were entered into the models separately. We analyzed data using IBM SPSS Statistics for Windows, version 27 (IBM Corp., Armonk, N.Y., USA). Statistical significance was determined at α of 0.05 (two-tailed).

## Results

Overall, 750 patients with suspected IS were recruited [17]. After exclusion of drop-outs (stroke mimics *n* = 41, withdrawal of consent *n* = 13) and patients without available hs-TnT levels (*n* = 153), 543 patients were included in the present analysis (Fig. 1). Mean age was 68.5 years (SD 13.5), and 337 patients (62%) were male. Median NIHSS score on admission was 3 (quartiles 1–5). Baseline characteristics are shown in Table 1. Patients without available troponin levels were older (71.5 vs 68.5 years, *p* = 0.014) but did not differ otherwise from included patients (see Additional file 1 Table I). Amongst the group of patients with available hs-TnT levels, ECG Holter monitoring was performed in 371 patients (68.3%). After exclusion of records not fulfilling prespecified quality criteria (see reasons for exclusion presented in Fig. 1), HRV analysis could be performed in 196 Holter ECG records (36.1%). Out of them, 42 patients (21.4%) had hs-TnT levels ≥14 ng/L. Patients undergoing HRV analysis were younger and had less comorbidities (see Additional file 1 Table II).

**Table 1:** Characterization of Study Population defined by troponin elevation after ischemic stroke

	All Patients (*n* = 543)	Patients with high-sensitive Troponin T ≥ 14 ng/L (*n* = 203)	Patients with high-sensitive Troponin T < 14 ng/L (*n* = 340)	*p*-value
Demographics				
Age (y), mean (SD)	68.5 (13.5)	76.3 (10.8)	63.8 (12.9)	< 0.001
Male sex, n (%)	337 (62.1)	132 (65.0)	205 (60.3)	0.272
Risk factors				
Atrial fibrillation, n (%)	128 (23.6)	87 (42.9)	41 (12.1)	< 0.001
Hypertension, n (%)	404 (75.0)	182 (90.5)	222 (65.6)	< 0.001
Diabetes mellitus, n (%)	109 (20.5)	54 (27.3)	55 (16.4)	0.003
Coronary artery disease, n (%)	91 (17.1)	51 (25.8)	40 (12.0)	< 0.001
Hyperlipidemia, n (%)	150 (27.6)	55 (27.1)	95 (27.9)	< 0.000
Clinically overt heart failure, n (%)	31 (6.0)	21 (11.4)	11 (3.4)	< 0.001
Cardiac dysfunction				
Systolic dysfunction, n (%)	53 (10.4)	33 (17.8)	20 (6.2)	< 0.001
Diastolic dysfunction, n (%)	117 (24.5)	71 (42.0)	46 (14.9)	< 0.001
Index event				
NIHSS, median (quartiles)	3 (1–5)	3 (2–6)	3 (1–5)	0.004
Insular stroke, n (%)	71 (13.1)	31 (15.3)	40 (11.7)	0.241
Etiology				< 0.001
Large artery atherosclerosis, n (%)	68 (12.5)	24 (11.8)	44 (12.9)	
Cardioembolic, n (%)	161 (29.7)	86 (42.4)	75 (22.1)	
Small artery occlusion, n (%)	76 (14.0)	24 (11.8)	52 (15.3)	
Other defined cause, n (%)	19 (3.5)	2 (1.0)	13/340 (3.8)	
Undefined, n (%)	219 (40.3)	67 (33.0)	152 (44.7)	
eGFR (mL/min/1.73 m^2^), mean (SD)	83.7 (19.5)	73.1 (21.4)	90 (15.2)	< 0.001
eGFR < 60 mL/min/1.73 m^2^, n (%)	68 (12.5)	56 (27.6)	12 (3.5)	< 0.001
High-sensitivity Troponin T (ng/L), median (quartiles)	10.9 (5.5–20.8)	25.3 (18.8–44.1)	6.75 (4.99–10.2)	
High-sensitivity C-reactive protein (mg/dL), median (quartiles)	0.26 (0.11–0.79)	0.52 (0.20–1.47)	0.17 (0.08–0.48)	< 0.001
Heart rate variability†				
Heart rate, mean (SD)†	69.5 (10.6)	71.7 (10.5)	69.0 (10.5)	0.138
SDNN (ms), mean (SD)†	112.9 (36.9)	106.6 (42.5)	114.6 (35.1)	0.265
RMSSD (ms), mean (SD)†	28.0 (14.7)	27.3 (17.1)	28.2 (14.1)	0.751
SDNN Index (ms), mean (SD)†	49.1 (19.0)	42.2 (18.4)	54. 1 (43.2)	0.008
SDANN (ms), mean (SD)†	97.3 (35.9)	95.1 (41.6)	97.9 (34.5)	0.656
Beginn Holter record (h after symptoms onset), median (quartiles)†	32 (20–53)	38 (21.5–57.5)	30.5 (18.3–52.8)	0.446

*NIHSS* National Institutes of Health Stroke Scale, *eGFR* estimated glomerular filtration rate, *SD* standard deviation, *h* hour. †Available for 196 patients

### Determinants of troponin elevation

Amongst 543 patients with available hs-TnT levels, troponin was detectable in 430 out of 543 (79%), and 203 patients (37%) had levels ≥14 ng/L. Troponin elevation was independently associated with older age (OR per year 1.05; 95% CI 1.02–1.08), male sex (OR 2.65; 95% CI 1.54–4.58), systolic dysfunction (OR 2.79; 95% CI 1.22–6.37), diastolic dysfunction in absence of systolic dysfunction (OR 2.29; 95% CI 1.29–4.02), atrial fibrillation (OR 2.3; 95% CI 1.25–4.23), decreasing eGFR (OR per 10 mL/min/1.73 m^2^ 0.71; 95% CI 0.61–0.84), and increasing hs-CRP levels (OR 1.48 per log-unit; 95% CI 1.22–1.79) (Table 2). Previous history of CAD, stroke severity, and insular lesion were not independently associated with hs-TnT ≥14 ng/L. The reported associations did not significantly change after adjusting for atherogenic cardiovascular risk factors (see Additional file 1 Table III). After adjustment for time point of sampling, the lower bound of the confidence interval for atrial fibrillation was 0.99 (see Additional file 1 Table IV).

**Table 2:** Determinants of elevated troponin levels in patients with ischemic stroke in multivariable logistic regression analysis

	Multivariable Logistic Regression OR (95% CI)
Age, per year	1.05 (1.02–1.08)
Male sex	2.65 (1.54–4.58)
Systolic dysfunction	2.79 (1.22–6.37)
Diastolic dysfunction (in absence of systolic dysfunction)	2.29 (1.29–4.02)
Pre-stroke history of coronary artery disease	1.57 (0.93–3.29)
Atrial fibrillation	2.30 (1.25–4.23)
Glomerular filtration rate, per 10 mL/min/1.73 m^2^	0.71 (0.61–0.84)
NIHSS, per point	1.03 (0.96–1.11)
High-sensitive C-reactive protein, per log-unit	1.48 (1.22–1.79)
Insular stroke	1.26 (0.59–2.70)

*OR* odds ratio. *CI* confidence interval, *NIHSS*: National Institutes of Health Stroke Scale

### Association of HRV with troponin elevation

In univariate analyses, SDNN index was significantly reduced in patients with hs-TnT ≥14 ng/L (42.2 vs. 54.1 ms, *p* = 0.008, OR 0.970; 95% CI 0.949–0.0992, see Table 1 and Additional file 1 Table V). However, this association became non-significant after adjustment for age (OR 0.982; 95% CI 0.960–1.005, see Additional file 1 Table V). We found no association of SDNN, RMSSD, and SDANN with hs-TnT ≥14 ng/L.

## Discussion

Our study reports determinants associated with elevated baseline troponin levels in a cohort of patients with acute IS undergoing extensive cardiac phenotyping. Here, we showed that hs-TnT elevation >99th percentile is independently associated with older age, male sex, decreasing eGFR, elevated hs-CRP, and cardiac disease (systolic dysfunction, diastolic dysfunction in the absence of systolic dysfunction or atrial fibrillation). We did not find an independent association between the investigated HRV time domain variables and increased hs-TnT. To the best of our knowledge, this is the first study investigating the association of reduced HRV with troponin elevation among IS patients.

Rates of elevated hs-TnT in our study are in line with previous publications in IS patients applying comparable kits, ranging between 30 and 60% [5, 8, 28]. In accordance with a previous study [8], age was independently associated with hs-TnT ≥14 ng/L. Reports lacking an association of age with increased troponin investigated either other assays (troponin I) [9–11] or a higher URL of hs-TnT [12]. Importantly, the definition of the upper 99th percentile used in this study (≥14 ng/L) is based on the assay’s validation study on a population of apparently healthy volunteers and blood donors (mean age 44 years, range 20–71 years) [25]. It is unclear, how well this URL applies to a general elderly population. A large population-based study (*n* = 19,501, age range 18–98 years) reported an URL for hs-TnT as high as 47.1 and 38.6 ng/L for men and women ≥70 years, even after exclusion of individuals with known cardiovascular disease [29]. While increasing troponin levels may indeed reflect subclinical myocardial injury in the elderly [30], caution is needed when interpreting the results of routine troponin testing in this population. The association of male sex with increased troponin in our cohort differs from a previous report on IS patients, probably explained by the use of a different kit with a different URL [9], but is in line with previous studies from the general population [29, 31]. This finding might be explained by a higher left ventricular mass in men, even after adjustment for body surface area [32].

We found a strong correlation of eGFR with hs-TnT ≥14 ng/L, in line with previous reports [8, 12]. Troponin appears to be catabolized in tissues with high metabolic rate, such as the kidney, and thus impaired clearance may lead to a higher baseline levels [1]. Importantly, experimental evidence suggests that renal clearance dominates at low levels of troponin T (e.g. patients with chronic cardiac disease) [33]. This might explain the lack of association of increased Troponin I with eGFR in other studies using higher upper reference limits (40 ng/L [9] and 200 ng/L [3]).

We found an association of both systolic and diastolic dysfunction with increased hs-TnT. Comparability with previous studies is limited, since most studies lacked structural and functional cardiac investigation [3, 5, 8, 11], excluded patients with systolic dysfunction [10], or echocardiographic data was available for < 60% of the population [9]. In previous reports, heart failure was frequently associated with increased troponin levels [8, 9]. However, the majority of IS patients with systolic or diastolic dysfunction are asymptomatic and therefore do not fulfill heart failure criteria [17]. Thus, the sole adjustment for heart failure, either self-reported or from medical records, insufficiently depicts the extent of existing cardiac dysfunction among IS patients. Possible explanations for the association of systolic dysfunction and troponin elevation are manifold. We have previously reported an association between pre-stroke CAD and systolic dysfunction [17]. Thus, both systolic dysfunction and troponin elevation might be correlates of previous myocardial ischemia. CAD is highly prevalent in IS patients [34] and approximately 3.5% of them will suffer an acute myocardial infarction during the index hospitalization [4]. However, only a quarter of IS patients undergoing coronary angiography presents a coronary culprit lesion [35] and troponin elevation is associated with poor prognosis even after exclusion of concurrent myocardial infarction [4]. We were not able to find an association of previous CAD with elevated troponin levels and < 40% of patients with systolic dysfunction in our cohort had a previous history of CAD [17]. Troponin elevation may also reflect a subclinical myocardial infarction occurring shortly before or after symptoms onset. In addition, experimental evidence suggests that IS might induce systolic dysfunction [36, 37]. Thus, systolic dysfunction and elevated troponin may be correlates of ongoing neurogenic myocardial injury. Alternatively, circulating troponin might indicate ongoing fibrosis in patients without known cardiac disease [31], which might explain the association of troponin (even <99th percentile) with systolic dysfunction in the general population [31]. Fibrosis is also involved in the development of diastolic dysfunction [38] and might explain the association we observed.

Nearly all patients with atrial fibrillation have detectable troponin levels and increasing values are associated with increasing risk of stroke [39]. Thus, IS patients might have increased troponin level at baseline correlating with increased baseline stroke risk. Furthermore, troponin release could be related to rapid ventricular response or the mechanical effects of fibrillation on the atria [40]. Further, atrial fibrillation may lead to coronary macro or microembolism, although this phenomenon seems to be rare [41]. This association barely missed statistical significance after adjustment for time point of blood sampling, thus suggests a potential role of time point of sampling and with the presence of elevated troponin after ischemic stroke.

Only one study has previously reported an association of an inflammation marker (tumor necrosis factor alpha) with troponin elevation [11], although its modest sample size and lack of adjustment for relevant confounders are major limitations. The association of the inflammation marker hs-CRP with increased troponin levels could reflect a pro-inflammatory state associated with traditional cardiovascular risk factors [42]. However, this association remained significant after adjustment for atherogenic cardiovascular risk factors. Alternatively, it could represent a stroke-induced immune response, which in an experimental study was associated with the development of systolic dysfunction [37].

Autonomic dysfunction with sympathetic overweight is another proposed mechanism to explain troponin elevation in IS patients [3]. We are aware of only one study investigating this hypothesis at the mechanistic level, showing an independent association of epinephrine (not norepinephrine) levels with increased troponin [3]. However, plasma epinephrine probably better reflects the adrenomedullary hormonal than the sympathetic noradrenergic system activation (reflected by plasma norepinephrine) [13]. Furthermore, epinephrine levels increase more markedly than norepinephrine to a wide range of stressors [13], which may limit their interpretability in conditions of environmental stress, such as a stroke unit. In our sensitivity analysis, only SDNN index was associated with elevated troponin levels in univariate analysis, although this association disappeared when adjusting for age. This finding was not unexpected, since SDNN index is the time domain variable most closely correlated with age, exhibiting a linearly declining pattern across the lifespan [43]. Thus, this association seems not to provide additional clinically relevant information.

Overall, our results suggest that troponin elevation in IS patients is predominantly associated with some traditional cardiovascular risk factors and symptomatic or asymptomatic cardiac disease. Thus, the prognostic value of troponin in IS patients probably reflects an increased baseline cardiovascular risk and may explain the inconsistent association of troponin with poor outcome found in a recent systematic review, which was more often non-significant when adjusted for other relevant cardiac prognostic factors (such as cardiac comorbidities or biomarkers) [7]. Our data does not support an association between reduced HRV and troponin elevation, at least among younger stroke patients with relatively few comorbidities. However, patients with low cardiovascular baseline risk represent a group where autonomic dysfunction could be especially important in explaining troponin elevation. Our data does not exclude an association of reduced HRV with troponin elevation in individuals with high cardiovascular baseline risk.

### Strengths and limitations

The major strength of our study is the assessment of the determinants of hs-TnT elevation within a large, prospective study of IS patients undergoing detailed and standardized cardiac examination. Our study has, however, limitations. First, this analysis was not prespecified and we analyzed the determinants of hs-TnT levels only at baseline. Thus, we might have missed transient troponin elevation during the hyperacute phase. However, previous results using the same hs-TnT assay do not suggest a clear dynamic during the first four days after IS [28]. We cannot extend our results to other troponin kits, since 99th percentiles of troponin I and T may not be biologically equivalent [29]. Second, this cohort consisted of mostly mild strokes, since patients with severe stroke are often unable to provide informed consent [44] and in the absence of a legal representative their recruitment is not possible during the acute phase. Therefore, the conclusions might not be extrapolated to more severely affected patients. Nonetheless, the primary analysis included a significant proportion of patients with insular involvement, a factor that has been repeatedly associated with cardiac complications. Third, we limited the analysis of HRV data to recordings fulfilling strict inclusion criteria, thus resulting in a smaller, healthier cohort. This limits the generalizability of our results. Further, this sensitivity analysis may lack power to detect subtle associations between time domain HRV variables and troponin elevation. Fourth, 24-hour ECG Holter records may be more suitable for cardiac risk stratification than detailed physiological investigation, since standardization of a 24-hour record is challenging [45]. However, the vast majority of records were obtained during the stay of patients at the stroke unit, thus providing rather standardized environmental conditions in terms of e.g. physical activity. Fifth, CAD was not systematically investigated in our cohort. However, previous results suggest that most IS patients with troponin elevation, even well above the 99th percentile, do not present an angiographic coronary culprit lesion [35]. Lastly, we do not report on patient’s outcomes. While a previous systematic review has shown that the association of troponin with poor functional outcome and mortality after ischemic stroke is mostly mediated by cardiac comorbidity [7], the prediction of major cardiovascular events represents a potentially relevant clinical application of troponin measurement after acute stroke [46], question that we will address in a separate study including a broader panel of cardiac biomarkers.

## Conclusions

Almost a third of patients with acute IS exhibited elevated troponin levels. Our data suggest that older age, male sex, clinical or subclinical cardiac disease, impaired renal function, and elevated hs-CRP are associated with increased troponin, while it did not support an association with reduced HRV. The clinical interpretation of elevated troponin levels must account for a broad spectrum of comorbidities and clinical characteristics. The pathophysiological relevance of autonomic dysfunction –especially in older, comorbid IS patients and in patients with severe stroke– and other markers of inflammation beyond hs-CRP in troponin elevation in IS patients as well the potential use of troponin to predict cardiovascular events after acute IS must be addressed in further studies.

## Supplementary Information

**Additional file 1: Appendix I.** Methods. **Appendix Table I.** Comparison of patients with and without available troponin measurement (“non-responder” analysis). **Appendix Table II.** Comparison of patients with and without available heart rate variability analysis (“non-responder” analysis). **Appendix Table III.** Determinants of elevated troponin levels in patients with ischemic stroke in multivariable logistic regression analysis after adjustment for cardiovascular risk factors. **Appendix Table IV.** Determinants of elevated troponin levels in patients with ischemic stroke in multivariable logistic regression analysis after adjustment for time point of blood sampling. **Appendix Table V.** Association of time domain variables of the heart rate variability with troponin elevation

## Data Availability

The data that support the findings of this study are available from the corresponding author upon reasonable request.

## Abbreviations

CAD: coronary artery disease
eGFR: estimated glomerular filtration rate
HRV: heart rate variability
Hs-CRP: high-sensitive C-reactive protein
Hs-TnT: high-sensitive troponin T
IS: ischemic stroke
SD: standard deviation
SDNN: standard deviation of normal-to-normal-beats
SDANN: standard deviation of the averages of normal-to-normal intervals for all 5-min segments for 24-h
SDNN index: mean of 5-min standard deviations of all normal-to-normal intervals for 24-h
RMSSD: root mean square of successive RR interval differences
URL: upper reference limit

## References

[1] Mair J, Lindahl B, Hammarsten O, Muller C, Giannitsis E, Huber K (2018). How is cardiac troponin released from injured myocardium?. Eur Heart J Acute Cardiovasc Care.

[2] Yaghi S, Chang AD, Ricci BA, Jayaraman MV, McTaggart RA, Hemendinger M (2018). Early elevated troponin levels after ischemic stroke suggests a cardioembolic source. Stroke.

[3] Barber M, Morton JJ, Macfarlane PW, Barlow N, Roditi G, Stott DJ (2007). Elevated troponin levels are associated with sympathoadrenal activation in acute ischaemic stroke. Cerebrovasc Dis.

[4] Wrigley P, Khoury J, Eckerle B, Alwell K, Moomaw CJ, Woo D (2017). Prevalence of positive troponin and echocardiogram findings and association with mortality in acute ischemic stroke. Stroke.

[5] Scheitz JF, Mochmann HC, Erdur H, Tutuncu S, Haeusler KG, Grittner U, et al. Prognostic relevance of cardiac troponin T levels and their dynamic changes measured with a high-sensitivity assay in acute ischaemic stroke: analyses from the TRELAS cohort. Int J Cardiol. 2014;177:886–93. 10.1016/j.ijcard.2014.10.036 25453407

[6] Powers WJ, Rabinstein AA, Ackerson T, Adeoye OM, Bambakidis NC, Becker K, et al. 2018 Guidelines for the early management of patients with acute ischemic stroke. Stroke. 2018;49:e46–e110. 10.1161/STR.0000000000000158 29367334

[7] Montellano FA, Ungethum K, Ramiro L, Nacu A, Hellwig S, Fluri F (2021). Role of blood-based biomarkers in ischemic stroke prognosis: a systematic review. Stroke.

[8] Faiz KW, Thommessen B, Einvik G, Brekke PH, Omland T, Ronning OM. Determinants of high sensitivity cardiac troponin T elevation in acute ischemic stroke. BMC Neurol. 2014;14:96. 10.1186/1471-2377-14-96 PMC4107722 24885286

[9] Ahn SH, Lee JS, Kim YH, Kim BJ, Kim YJ, Kang DW (2017). Prognostic significance of troponin elevation for long-term mortality after ischemic stroke. J Stroke.

[10] Ahn SH, Kim YH, Shin CH, Lee JS, Kim BJ, Kim YJ, et al. Cardiac vulnerability to cerebrogenic stress as a possible cause of troponin elevation in stroke. J Am Heart Assoc. 2016;5(10):e004135. 10.1161/JAHA.116.004135. Published online 2016 Oct 6. 10.1161/JAHA.116.004135 PMC5121511 27792642

[11] Christensen H, Johannesen HH, Christensen AF, Bendtzen K, Boysen G. Serum cardiac troponin i in acute stroke is related to serum cortisol and TNF-alpha. Cerebrovasc Dis. 2004;18:194–9. 10.1159/000079941 15273434

[12] Scheitz JF, Endres M, Mochmann HC, Audebert HJ, Nolte CH (2012). Frequency, determinants and outcome of elevated troponin in acute ischemic stroke patients. Int J Cardiol.

[13] Goldstein DS (2010). Catecholamines 101. Clin Auton Res.

[14] De Raedt S, De Vos A, De Keyser J (2015). Autonomic dysfunction in acute ischemic stroke: an underexplored therapeutic area?. J Neurol Sci.

[15] Heart rate variability (1996). Standards of measurement, physiological interpretation, and clinical use. Task force of the european society of cardiology and the north american society of pacing and electrophysiology. Eur Heart J.

[16] Sposato LA, Hilz MJ, Aspberg S, Murthy SB, Bahit MC, Hsieh CY (2020). Post-stroke cardiovascular complications and neurogenic cardiac injury: Jacc state-of-the-art review. J Am Coll Cardiol.

[17] Heuschmann PU, Montellano FA, Ungethum K, Rucker V, Wiedmann S, Mackenrodt D, et al. Prevalence and determinants of systolic and diastolic cardiac dysfunction and heart failure in acute ischemic stroke patients: the SICFAIL study. ESC Heart Fail. 2021;8:1117–29. 10.1002/ehf2.13145 PMC8006617 33350167

[18] Hatano S (1976). Experience from a multicentre stroke register: a preliminary report. Bull World Health Organ.

[19] Morbach C, Gelbrich G, Breunig M, Tiffe T, Wagner M, Heuschmann PU, et al. Impact of acquisition and interpretation on total inter-observer variability in echocardiography: results from the quality assurance program of the STAAB cohort study. Int J Cardiovasc Imaging. 2018;34:1057–65. 10.1007/s10554-018-1315-3 29445974

[20] Lang RM, Badano LP, Mor-Avi V, Afilalo J, Armstrong A, Ernande L (2015). Recommendations for cardiac chamber quantification by echocardiography in adults. Eur Heart J Cardiovasc Imaging.

[21] Nagueh SF, Smiseth OA, Appleton CP, Byrd BF, Dokainish H, Edvardsen T (2016). Recommendations for the evaluation of left ventricular diastolic function by echocardiography. Eur Heart J Cardiovasc Imaging.

[22] Luers C, Edelmann F, Wachter R, Pieske B, Mende M, Angermann C (2017). Prognostic impact of diastolic dysfunction in systolic heart failure-a cross-project analysis from the german competence network heart failure. Clin Cardiol.

[23] Ponikowski P, Voors AA, Anker SD, Bueno H, Cleland JGF, Coats AJS, et al. 2016 ESC guidelines for the diagnosis and treatment of acute and chronic heart failure. Eur Heart J. 2016;37:2129–200.

[24] Geiger J, Both S, Kircher S, Neumann M, Rosenwald A, Jahns R (2018). Hospital-integrated biobanking as a service – the interdisciplinary bank of biomaterials and data wuerzburg (ibdw). Open J Bioresources.

[25] Giannitsis E, Kurz K, Hallermayer K, Jarausch J, Jaffe AS, Katus HA. Analytical validation of a high-sensitivity cardiac troponin T assay. Clin Chem. 2010;56:254–61. 10.1373/clinchem.2009.132654 19959623

[26] Levey AS, Stevens LA, Schmid CH, Zhang YL, Castro AF, Feldman HI (2009). A new equation to estimate glomerular filtration rate. Ann Intern Med.

[27] Irfan A, Twerenbold R, Reiter M, Reichlin T, Stelzig C, Freese M, et al. Determinants of high-sensitivity troponin T among patients with a noncardiac cause of chest pain. Am J Med. 2012;125(491–498):e491. 10.1016/j.amjmed.2011.10.031 22482847

[28] Jensen JK, Ueland T, Aukrust P, Antonsen L, Kristensen SR, Januzzi JL, et al. Highly sensitive troponin T in patients with acute ischemic stroke. Eur Neurol. 2012;68:287–93. 10.1159/000341340 23051820

[29] Welsh P, Preiss D, Shah ASV, McAllister D, Briggs A, Boachie C, et al. Comparison between high-sensitivity cardiac troponin T and cardiac troponin I in a large general population cohort. Clin Chem. 2018;64:1607–16. 10.1373/clinchem.2018.292086 PMC6398571 30126950

[30] Shah ASV, Sandoval Y, Noaman A, Sexter A, Vaswani A, Smith SW (2017). Patient selection for high sensitivity cardiac troponin testing and diagnosis of myocardial infarction: prospective cohort study. BMJ.

[31] Seliger SL, Hong SN, Christenson RH, Kronmal R, Daniels LB, Lima JAC, et al. High-sensitive cardiac troponin t as an early biochemical signature for clinical and subclinical heart failure: MESA (Multi-Ethnic Study of Atherosclerosis). Circulation. 2017;135:1494–505. 10.1161/CIRCULATIONAHA.116.025505 PMC5401621 28159799

[32] Salton CJ, Chuang ML, O'Donnell CJ, Kupka MJ, Larson MG, Kissinger KV (2002). Gender differences and normal left ventricular anatomy in an adult population free of hypertension. A cardiovascular magnetic resonance study of the Framingham heart study offspring cohort. J Am Coll Cardiol.

[33] Friden V, Starnberg K, Muslimovic A, Ricksten SE, Bjurman C, Forsgard N, et al. Clearance of cardiac troponin T with and without kidney function. Clin Biochem. 2017;50:468–74. 10.1016/j.clinbiochem.2017.02.007 28193484

[34] Amarenco P, Lavallee PC, Labreuche J, Ducrocq G, Juliard JM, Feldman L (2011). Prevalence of coronary atherosclerosis in patients with cerebral infarction. Stroke.

[35] Mochmann HC, Scheitz JF, Petzold GC, Haeusler KG, Audebert HJ, Laufs U, et al. Coronary angiographic findings in acute ischemic stroke patients with elevated cardiac troponin: the troponin elevation in acute ischemic stroke (TRELAS) study. Circulation. 2016;133:1264–71. 10.1161/CIRCULATIONAHA.115.018547 26933082

[36] Bieber M, Werner RA, Tanai E, Hofmann U, Higuchi T, Schuh K (2017). Stroke-induced chronic systolic dysfunction driven by sympathetic overactivity. Ann Neurol.

[37] Yan T, Chen Z, Chopp M, Venkat P, Zacharek A, Li W (2020). Inflammatory responses mediate brain-heart interaction after ischemic stroke in adult mice. J Cereb Blood Flow Metab.

[38] Moreo A, Ambrosio G, De Chiara B, Pu M, Tran T, Mauri F (2009). Influence of myocardial fibrosis on left ventricular diastolic function: noninvasive assessment by cardiac magnetic resonance and echo. Circ Cardiovasc Imaging.

[39] Hijazi Z, Wallentin L, Siegbahn A, Andersson U, Alexander JH, Atar D, et al. High-sensitivity troponin T and risk stratification in patients with atrial fibrillation during treatment with apixaban or warfarin. J Am Coll Cardiol. 2014;63:52–61. 10.1016/j.jacc.2013.07.093 24055845

[40] Kaura A, Arnold AD, Panoulas V, Glampson B, Davies J, Mulla A, et al. Prognostic significance of troponin level in 3121 patients presenting with atrial fibrillation (the NIHR Health Informatics Collaborative TROP-AF study). J Am Heart Assoc. 2020;9:e013684. 10.1161/JAHA.119.013684 PMC7428631 32212911

[41] Shibata T, Kawakami S, Noguchi T, Tanaka T, Asaumi Y, Kanaya T (2015). Prevalence, clinical features, and prognosis of acute myocardial infarction attributable to coronary artery embolism. Circulation.

[42] Lindsberg PJ, Grau AJ (2003). Inflammation and infections as risk factors for ischemic stroke. Stroke.

[43] Umetani K, Singer DH, McCraty R, Atkinson M (1998). Twenty-four hour time domain heart rate variability and heart rate: relations to age and gender over nine decades. J Am Coll Cardiol.

[44] Hotter B, Ulm L, Hoffmann S, Katan M, Montaner J, Bustamante A (2017). Selection bias in clinical stroke trials depending on ability to consent. BMC Neurol.

[45] Malik M (2011). Assessment of cardiac autonomic regulation. Ann Noninvasive Electrocardiol.

[46] Murthy SB (2021). Troponin elevation after ischemic stroke and future cardiovascular risk: is the heart in the right place?. J Am Heart Assoc.

