# Supplementary material for: Cardiac dysfunction and high-sensitive C-reactive protein are associated with troponin T elevation in ischemic stroke: insights from the SICFAIL study
Source: BMC Neurol. 2022 Dec 31;22:511. doi: 10.1186/s12883-022-03017-1 (PMC9804953; doi:10.1186/s12883-022-03017-1)
Supplement: Supplementary file 1 — Additional file 1: Appendix I. Methods. Appendix Table I. Comparison of patients with and without available troponin measurement (“non-responder” analysis). Appendix Table II. Comparison of patients with and without available heart rate variability analysis (“non-responder” analysis). Appendix Table III. Determinants of elevated troponin levels in patients with ischemic stroke in multivariable logistic regression analysis after adjustment for cardiovascular risk factors. Appendix Table IV. Determinants of elevated troponin levels in patients with ischemic stroke in multivariable logistic regression analysis after adjustment for time point of blood sampling. Appendix Table V. Association of time domain variables of the heart rate variability with troponin elevation [file 12883_2022_3017_MOESM1_ESM.docx]

**Supplemental Material**

**Appendix I** Methods

**Table I**. Comparison of patients with and without available troponin measurement (“non-responder” analysis)

**Table II**. Comparison of patients with and without available heart rate variability analysis (“non-responder” analysis)

**Table III** Determinants of elevated troponin levels in patients with ischemic stroke in multivariable logistic regression analysis after adjustment for cardiovascular risk factors

**Table IV** Determinants of elevated troponin levels in patients with ischemic stroke in multivariable logistic regression analysis after adjustment for time point of blood sampling

**Table V** Association of time domain variables of the heart rate variability with troponin elevation

**Appendix I**

Methods

*Diagnostic Workup*

All patients underwent neuroimaging (computed tomography or magnetic resonance imaging), vascular imaging (Doppler, CT and/or MRI angiography), 12-lead electrocardiogram on admission, and ECG-monitoring at the stroke unit or intensive care unit.

*Baseline investigation*

Hypertension was defined as self-reported history of hypertension or intake of antihypertensive medication. Coronary artery disease was defined as self-reported history of previous myocardial infarction or history of angina. Atrial fibrillation was defined according to the medical records of the index event or a new diagnosis of atrial fibrillation during the index stay. Diabetes mellitus was defined as self-reported history of diabetes, glycosylated haemoglobin A1c ≥ 6.5% or intake of antidiabetic medication. Hypercholesterolemia was defined according to self-reported previous history of the disease.

*Biomarker measurement*

High-sensitive Troponin T (hs-TnT), high-sensitive C-reactive protein (hs-CRP) and creatinine were measured from frozen serum samples at a single time point at the end of study by a technician blinded to all clinical data using the assays Elecsys Troponin T high sensitive STAT (Roche Diagnostics, Mannheim, Germany), Cardiac C-Reactive Protein (Latex) High- Sensitive, and Creatinine plus ver. 2 (Roche Diagnostics, Mannheim, Germany). Because the lower limit of detection of the hs-TnT assay is 5 ng/L, nondetectable hs-TnT levels are presented as 4.99 ng/L. Low density lipoprotein was measured as part of the standard diagnostic workup the morning after admission using the assay LDL-Cholesterol plus 2^nd^ generation (Roche Diagnostics, Mannheim, Germany).

**Table I**. Comparison of patients with and without available troponin measurement (“non-responder” analysis)

|  | Patients with available high-sensitive Troponin T (n=543) | Patients without available high-sensitive Troponin T (n=153) | p-value |
| --- | --- | --- | --- |
| Age (years), mean (SD) | 68.5 (13.6) | 71.5 (12.6) | 0.014 |
| Male, n (%) | 337 (62.1) | 92 (60.1) | 0.664 |
| NIHSS, median (quartiles) | 3 (1–5) | 3 (1–5) | 0.896 |
| Systolic dysfunction, n (%) | 53 (10.4) | 9 (6.7) | 0.190 |
| Diastolic dysfunction in absence of systolic dysfunction, n (%) | 117 (24.5) | 37 (29.1) | 0.290 |
| Atrial fibrillation, n (%) | 128 (23.6) | 36 (23.5) | 0.991 |
| Insular stroke, n (%) | 71 (13.1) | 21 (13.7) | 0.834 |

SD: standard deviation.

**Table II**. Comparison of patients with and without available heart rate variability analysis (“non-responder” analysis)

|  | Patients with available heart rate variability analysis T (n=196) | Patients without available heart rate variability analysis T (n=153) | p-value |
| --- | --- | --- | --- |
| Age (years), mean (SD) | 64.1 (12.7) | 71.1 (13.1) | <0.001 |
| Male, n (%) | 123 (62.8) | 306 (61.2) | 0.704 |
| NIHSS, median (quartiles) | 2 (1–4.5) | 3 (1–5) | 0.056 |
| Systolic Dysfunction n (%) | 12 (6.4) | 50 (10.9) | 0.077 |
| Diastolic dysfunction in absence of systolic dysfunction n (%) | 32 (17.7) | 122 (28.8) | 0.004 |
| Clinically overt heart failure, n (%) | 10 (5.3) | 25 (5.5) | 0.950 |
| Atrial fibrillation n (%) | 8 (4.1) | 156 (31.2) | <0.001 |
| Insular lesion, n (%) | 24 (12.2) | 68 (13.6) | 0.635 |
| High-sensitive Troponin T (ng/L), median (quartiles) | 7.55 (4.99–13.0) | 13.20 (7.2–24.5) | <0.001 |
| High-sensitive C-reactive protein (mg/dL), median (quartiles) | 0.16 (0.07–0.49) | 0.34 (0.14–1.01) | <0.001 |
| Glomerular filtration rate (mL/min/1.73 m^2^), mean (SD) | 87.6 (18.7) | 81.5 (19.6) | <0.001 |
| Glomerular filtration rate <60 mL/min/1.73 m^2^, n (%) | 18 (9.2) | 348 (14.4) | 0.079 |

SD: standard deviation.

Table III Determinants of elevated troponin levels in patients with ischemic stroke in multivariable logistic regression analysis after adjustment for cardiovascular risk factors

|  | Multivariable  Logistic  Regression†  OR (95 % CI) |
| --- | --- |
| Age, per year | 1.05 (1.02–1.08) |
| Male sex | 2.94 (1.62–5.32) |
| Systolic dysfunction | 3.05 (1.30–7.20) |
| Diastolic dysfunction (in absence of systolic dysfunction) | 2.21 (1.24–3.96) |
| Pre-stroke history of coronary artery disease | 1.65 (0.83–3.31) |
| Atrial fibrillation | 2.34 (1.24–4.41) |
| Glomerular filtration rate, per 10 mL/min/1.73 m^2^ | 0.73 (0.62–0.86) |
| NIHSS, per point | 1.03 (0.95–1.11) |
| High-sensitive C-reactive protein, per log-unit | 1.38 (1.12–1.69) |
| Insular stroke | 1.14 (0.52–2.51) |
| Diabetes mellitus | 1.26 (0.71–2.22) |
| Hypertension |  |
| No hypertension | Reference |
| Treated hypertension | 0.61 (0.29–1.27) |
| Untreated hypertension | 1.18 (0.45–3.08) |
| Smoking |  |
| No previous smoking | Reference |
| Previous smoker, current cessation | 0.69 (0.38–1.26) |
| Current smoker | 0.70 (0.31–1.55) |
| Pre-stroke history of hyperlipidaemia | 0.67 (0.38–1.18) |
| Low density lipoprotein, per 1 mg/dL increase | 1.00 (0.99–1.01) |

OR: odds ratio. CI: confidence interval, NIHSS: National Institutes of Health Stroke Scale

Table IV Determinants of elevated troponin levels in patients with ischemic stroke in multivariable logistic regression analysis after adjustment for time point of blood sampling

|  | Multivariable  Logistic  Regression  OR (95 % CI) |
| --- | --- |
| Age, per year | 1.06 (1.03–1.09) |
| Male sex | 2.88 (1.63–5.08) |
| Systolic dysfunction | 3.02 (1.24–7.26) |
| Diastolic dysfunction (in absence of systolic dysfunction) | 2.26 (1.4–4.83) |
| Pre-stroke history of coronary artery disease | 1.59 (0.80–3.17) |
| Atrial fibrillation | 1.90 (0.99–3.56) |
| Glomerular filtration rate, per 10 mL/min/1.73 m^2^ | 0.72 (0.61–0.85) |
| NIHSS, per point | 1.03 (0.95–1.11) |
| High-sensitive C-reactive protein, per log-unit | 1.46 (1.18–1.79) |
| Insular stroke | 0.98 (0.44–2.15) |
| Day to sampling | 0.78 (0.62–0.999) |

OR: odds ratio. CI: confidence interval, NIHSS: National Institutes of Health Stroke Scale

Table V. Association of time domain variables of the heart rate variability with troponin elevation

|  | Univariable Logistic  Regression OR (95 % CI) | Model 1 OR (95 % CI) | Model 2 OR (95 % CI) |
| --- | --- | --- | --- |
| SDNN, per ms | 0.994 (0.984–1.004) | 0.995 (0.985–1.006) | 0.996 (0.985–1.007) |
| SDANN, per ms | 0.998 (0.988–1.008) | 0.998 (0.988–1.009) | 0.999 (0.988–1.010) |
| SDNN Index, per ms | 0.970 (0.949–0.0992) | 0.982 (0.960–1.005) | 0.983 (0.961–1.006) |
| RMSSD, per ms | 0.996 (0.972–1.020) | 1.000 (0.974–1.027) | 1.003 (0.976–1.031) |

OR: odds ratio. CI: confidence interval, SDNN: standard deviation (SD) of normal‐to‐normal‐beats, SDANN: SD of the averages of normal-to-normal intervals for all 5‐min segments for 24 h, SDNN Index: mean of 5‐min SDs of all normal-to-normal intervals for 24 h, RMSSD: root mean square of successive RR interval differences (RMSSD). Model 1: Adjusted for age. Model 2. Adjusted for age and eGFR.
